# Application of Taguchi Design and Response Surface Methodology for Improving Conversion of Isoeugenol into Vanillin by Resting Cells of *Psychrobacter* sp. CSW4

**Published:** 2013

**Authors:** Morahem Ashengroph, Iraj Nahvi, Jahanshir Amini

**Affiliations:** a *Department of Biology and Biotechnology, Faculty of Sciences, University of Kurdistan, P.O. Box 416, Sanandaj, Iran.*; b *Department of Microbiology, School of Biology, University College of Science, University of Isfahan, Isfahan, Iran.*; c *Department of Plant Protection, Faculty of Agriculture, University of Kurdistan, P.O. Box 416, Sanandaj, Iran. *

**Keywords:** Bioconversion process parameters, Vanillin production, *Psychrobacter *sp. CSW4, Optimization, Taguchi design, Response surface methodology

## Abstract

For all industrial processes, modelling, optimisation and control are the keys to enhance productivity and ensure product quality. In the current study, the optimization of process parameters for improving the conversion of isoeugenol to vanillin by *Psychrobacter *sp. CSW4 was investigated by means of Taguchi approach and Box-Behnken statistical design under resting cell conditions. Taguchi design was employed for screening the significant variables in the bioconversion medium. Sequentially, Box-Behnken design experiments under Response Surface Methodology (RSM) was used for further optimization. Four factors (isoeugenol, NaCl, biomass and tween 80 initial concentrations), which have significant effects on vanillin yield, were selected from ten variables by Taguchi experimental design. With the regression coefficient analysis in the Box-Behnken design, a relationship between vanillin production and four significant variables was obtained, and the optimum levels of the four variables were as follows: initial isoeugenol concentration 6.5 g/L, initial tween 80 concentration 0.89 g/L, initial NaCl concentration 113.2 g/L and initial biomass concentration 6.27 g/L. Under these optimized conditions, the maximum predicted concentration of vanillin was 2.25 g/L. These optimized values of the factors were validated in a triplicate shaking flask study and an average of 2.19 g/L for vanillin, which corresponded to a molar yield 36.3%, after a 24 h bioconversion was obtained. The present work is the first one reporting the application of Taguchi design and Response surface methodology for optimizing bioconversion of isoeugenol into vanillin under resting cell conditions.

## Introduction

Vanillin is of major interest for the flavour and fragrance industry. It has been used as a flavoring agent in foods, perfumes, beverages, pharmaceuticals and medical industries (1). Two main approaches for the production of natural vanillin are direct extraction from botanical sources and microbial bioconversion. Considering the various applications of vanillin and increasing consumer-led demand for natural vanillin and also due to the fact that the extraction from botanic sources is very time consuming, expensive and does not satisfy the worldwide demand, alternative methods for its production are being constantly explored (2). One of the biotechnological methods for the production of natural vanillin is the conversion of isoeugenol. As useful precursor for a more economical vanillin production, natural isoeugenol extracted from cheap plant-derived phenylpropanoids has been widely investigated using microbial and enzymatic bioconversions (3). However, relatively low yields of vanillin were obtained for the biotransformation of isoeugenol due to the fact that the vanillin produced was subsequently further converted into undesirable end-products such as vanillyl alcohol and/or vanillic acid as well as because of isoeugenol toxicity and insolubility. Several methods have been developed and applied to improve the vanillin yield from biotransformation of isoeugenol by using different strategies including cell free extract (4), addition of adsorbent resin (5), resting cell strategy (6-8), and addition of organic solvents such as DMSO (6). The highest yield of natural vanillin obtained from conversion of isoeugenol (71%) is reported by Yamada *et al. *(6). However, since this process involved a toxic organic solvent, it was not usable for the commercial natural vanillin production. Process conditions optimization are essentially required to improve conversion of isoeugenol into vanillin. Traditional one-factor-at-time (OFAT) experiments frequently fail to identify the variables that give rise to the optimum response because the effects of parameter interactions are overlooked in such procedures (9). Moreover, it requires a very large number of experiments to be done, which would be very expensive and time consuming. An alternative strategy is the use of statistical approaches, which are the powerful and useful tools for process optimization studies in biotechnology (10). Taguchi design is an efficient optimization tool in preliminary studies to screen parameters that have significant effects on the production. A number of reports have been published regarding the application of the Taguchi approach in the field of biotechnology (11-13). As an efficient tool in the statistical design of experiments, Response Surface Methodology is a collection of mathematical and statistical techniques useful for modeling and analysis of problems, in which a response of interest is influenced by several input variables and the objective is to optimize this response (14). Response Surface Methods are designs and models for working with continuous treatments when finding the optimal or when describing the response is the goal (15). In recent years, RSM has been extensively applied for the optimization of multiple variables in many bioprocesses and showed satisfactory results (16-21). There are, however, a few reports regarding the application of response surface methodology for improving the conversion of ferulic acid substrate into vanillin product (22, 23). So far, there is no report on the application of RSM as a tool to study and optimize conditions for enhanced production of vanillin via conversion of isoeugenol substrate. The principal aim of the current investigation was to optimize the different ingredients of defined media for improving conversion of isoeugenol into vanillin by the native isolated *Psychrobacter *sp. strain CSW4 under resting cell conditions. Firstly, the Taguchi experimental design was used to screen significant variables. Box-Behnken design of response surface methodology was applied in the second step to determine the optimum levels of the factors that significantly influence the isoeugenol bioconversion rate and yield of vanillin.

## Experimental

*Microorganism and chemicals*

*Psychrobacter *sp. CSW4 (GeneBank accession no. HQ111515), a local moderately halotolerant bacterial strain, with potential conversion of isoeugenol into vanillin was employed in the current study, which was screened in our previous publication (8). It was preserved in 15% glycerol and stored at -20 ºC. The culture was grown by regular subculturing in saline LB medium (% w/v) [casein peptone 1%, yeast extract 0.5%, NaCl 8%, pH 7.0] and it was then stored at 4 ºC. The strain was deposited at the College of Biological Sciences, Kurdistan University, Sanadaj, Iran. All the chemicals used were of analytical reagent grade and were obtained from Sigma-Aldrich Company. Ltd., United Kingdom and Merck Eurolab (Darmstadt, Germany). 

*Resting cell conversion for production of vanillin*

*Psychrobacter *sp. CSW cells were cultured in 250 mL flasks containing 50 mL LB medium supplemented with 80 g/L NaCl for 15 h (mid-log growth phase), then 0.5 g/L of isoeugenol was added to induce the enzymes (24). The cells were performed on a rotary shaker with a speed of 200 rpm at 28°C. After an additional 15 h incubation (the late exponenterial growth phase ,30-h), the cultures were collected by centrifugation (3,000×g for 20 min at 4°C), washed with saline/phosphate buffer (disodium/potassium phosphate buffer (100 mM), 80 g/L NaCl, pH 7.0) and resuspended with the same phosphate buffer. The pelleted cells were reconstituted in the same buffer and used for all experiments as resting cells. Resting cell conversion was undertaken at 28 °C and 200 rpm in 250 mL flasks containing 50 mL saline phosphate buffer. The cell growth of *Psychrobacter *sp. strain CSW4 was estimated turbidimetrically at 600 nm and 0.48 g/L dry cell weight was equivalent to 1.0 unit of optical density at 600 nm. Vanillin obtained from isoeugenol bioconversion in *Psychrobacter *sp. CSW4 cultures was qualitatively analyzed with High Performance Liquid Chromatography (HPLC) as described by Ashengroph *et al. *(8). 

*Screening of significant factors by Taguchi design approach*

The Taguchi screening design was conducted to evaluate the relative importance of factors with respect to their main effects for vanillin production under resting cells of *Psychrobacter *sp. CSW4. For the selection of significant factors for improving conversion of isoeugenol to vanillin, a standard orthogonal array L12 with 11 degrees of freedom was applied to screen ten factors at two levels, a higher (+) and a lower (–) level as shown in Tables 1 and 2. 

**Table 1:** Assigned concentrations of factors at different levels in Taguchi design for vanillin production by resting cells of *Psychrobacter *sp. strain CSW4

**No.**	**Factor (g/L) **	**Low level (-)**	**High level (+)**
1	Isoeugenol	1	5
2	Dry Biomass	2.5	5
3	Tween 20	0	0.5
4	Tween 80	0	0.5
5	Glycerol	0	0.1
6	Yeast extract	0	0.1
7	Trypton	0	0.1
8	Cu^+2^	0	0.05
9	Zn^+2^	0	0.05
10	NaCl	0	50

**Table 2:** Taguchi experimental design matrix for screening of various factors for vanillin production using 12 trials

**Trial**	**Isoeugenol**	**Biomass**	**Tween 20**	**Tween 80**	**Glycerol**	**Yeast extract**	**Trypton**	**Cu** ^+2^	**Zn** ^+2^	**NaCl**	**Mean vanillin (g/L)**
1	-	-	-	-	-	-	-	-	-	-	0.066
2	-	-	-	-	-	+	+	+	+	+	0.324
3	-	-	+	+	+	-	-	-	+	+	0.392
4	-	+	-	+	+	-	+	+	-	-	0.512
5	-	+	+	-	+	+	-	+	-	+	0.501
6	-	+	+	+	-	+	+	-	+	-	0.391
7	+	-	+	+	-	-	+	+	-	+	1.02
8	+	-	+	-	+	+	+	-	-	-	0.379
9	+	-	-	+	+	+	-	+	+	-	0.466
10	+	+	+	-	-	-	-	+	+	-	0.521
11	+	+	-	+	-	+	-	-	-	+	1.08
12	+	+	-	-	+	-	+	-	+	+	0.977

The main effect of the tested variable was determined by using the following equation:

*E*_xi_= 2 (Σ_Mi__+ _- *Σ*_Mi__-_)/*N                     *(Equation 1) 

Where, *E(Xi) *is the concentration effect of the tested variable, *Mi*+ and *Mi*− are the vanillin yields from the trials where the variable (*Xi*) measured was present at high and low concentrations, respectively and *N *is the number of trials (25). A design of a total of 12 trials was generated and the bioconversion experiments were performed in 250 mL shake flasks (28 °C, 200 rpm) containing 50 mL of designed media, which were prepared by varying the concentration (g/L) of selected factors. After 24 h of bioconversion time, the amount of vanillin formed in the reaction mixture was evaluated by HPLC, which is presented as the mean value of three individual assays (Table 2). Analysis of data was carried out using the analysis of variance (ANOVA) method. All calculations were developed using Qualitek-4 software (Nutek, Inc., Bloomfi eld, MI, USA). 

*Optimization of screened variables by response surface methodology*

The screened variables were optimized by the Response surface methodology (RSM), using a Box-Behnken design (26) to enhance the yield of vanillin. Box-Behnken design only has three levels (low, medium, and high, coded as -1, 0, and +1) and needs fewer experimental runs and less time. Additionally, it is more effective and easier technique to arrange and interpret in comparison with others (27). A total number of 27 trials including three center points were employed. All the experiments were done independently in triplicates and the average yields of vanillin were presented as the response. The coded and uncoded values of the variables levels are listed in Table 4. The arrangement and results of the Box-Behnken design are shown in Table 5. The statistical software, Design Expert (Version 7, State-Ease Inc, USA) was applied for the Box-Behnken experimental design, regression analysis of the experimental data, quadratic model buildings and also to plot three-dimensional response surface graphs. Statistical analysis of the models was used to evaluate the analysis of variance (ANOVA). The quality of fit the second-order polynomial model equation was judged statistically via the coefficient of determination R^2^ and the adjusted R^2^. The fitted polynomial equation was then expressed in terms of three-dimensional surface plots to evaluate the relationship between the responses and visualize the interaction between the variables utilized in the study. The point optimization method was conducted in order to optimize the level of each variable for maximum response. The combination of different optimized variables, which yielded the maximum response was determined in an attempt to verify the validity of the model (28). 

## Results and Discussion

*Screening and Selection of Important factors by Taguchi design*

**Figure 1:**
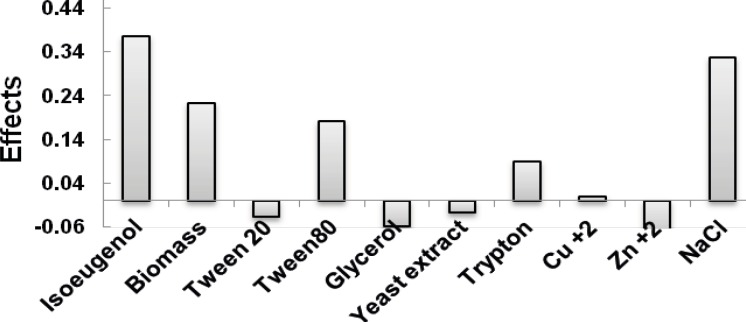
Effect of various factors on vanillin yield

For screening purpose, the Taguchi experimental design (ten-factor-two-level factorial design) was applied to identify the significant factors that influenced the conversion of isoeugenol to vanillin by the resting cells of *Psychrobacter *sp. CSW4. In this case, ten variables including isoeugenol, tryptone, yeast extract, glycerol, tween 20, tween 80, copper and zinc ions, biomass (dry weight) and sodium chloride concentrations were screened in 12 experiments by the Taguchi design (Table 1) and were chosen for the study. All trials were performed in triplicates and the average of observed determined vanillin concentrations for the 12 runs is shown in Table 2. The results show that the amount of vanillin produced ranged from 0.066 to 1.08 g/L corresponding to the combined influence of the ten variables in their specific ranges. The main effect of each variable on vanillin yield is shown in Figure 1. It is apparent from the Figure that, the effects of the ten individual variables in the order are as follows: Isoeugenol> NaCl> Biomass> Tween 80> Tryptone> Cu^+2^> yeast extract> Tween 20> glycerol> Zn^+2^. The data obtained from Taguchi experimental design on vanillin production were subjected to analysis of variance (ANOVA) as shown in Table 3. 

**Table 3:** Analysis of Variance (ANOVA) of Taguchi experiments results. The % column of the ANOVA indicates the influence of each factor on vanillin production. Other/error refer to experimental error, if any

**Before polling**													
**Serial no.**	**Factors**	**DOF**	**Sums of squares**	**Variance**	**F Ratio**	**Pure sum**	**Percentage**	**DOF**	**Sums of squares**	**Variance**	**F Ratio**	**Pure Sum**	**Percentage**
1	Isoeugenol	1	0.423	0.423	115.188	0.419	39.606	1	0.423	0.423	63.361	0.416	39.347
2	Biomass	1	0.147	0.147	40.226	0.144	13.605	1	0.147	0.147	22.127	0.141	13.33
3	Tween 20	1	0.003	0.003	1.077	0	0.026	(1)	(0.003)		POOLED	CL*=NC●	
4	Tween 80	1	0.1	0.1	27.234	0.096	9.099	1	0.1	0.1	14.98	0.093	8.821
5	Glycerol	1	0.002	0.002	0.67	0	0	(1)	(0.002)		POOLED	CL*=NC●	
6	Yeast extract	1	0.01	0.01	2.777	0.006	0.616	(1)	(0.01)		POOLED	CL=79.22 %	
7	Trypton	1	0.027	0.027	7.47	0.023	2.244	1	0.027	0.027	4.109	0.02	1.961
8	Cu^+2^	1	0	0	0.07	0	0	(1)	(0)		POOLED	CL*=NC●	
9	Zn^+2^	1	0.019	0.019	5.311	0.015	1.495	(1)	(0.019)		POOLED	CL=93.38 %	
10	NaCl	1	0.32	0.32	87.276	0.317	29.925	1	0.32	0.32	48.008	0.314	29.66
Other/error		1	0.003	0.003			3.384	6	0.039	0.006	6.881		
Total		11	1.059				100.00%	11	1.059				

ANOVA was employed to test the statistical factors and determine of significant parameters. To reduce the error term, the ANOVA data for vanillin production were improved by pooling the data until the degrees of freedom were reduced to close to half of the total; the data are exhibited in Table 3 under ‘After pooling’. From the results obtained, it was found that the effects of isoeugenol, NaCl, Biomass and tween 80 had confidence levels >99% and were considered to significantly influence vanillin production with this microorganism. The optimal conditions of these four ingredients needed to be further measured by the Box-Behnken design. The variable of tryptone that had confidence level above 95% were not included in the next optimization experiment, but was used in all experiments as a co-substrate at its high level (+) for the positively contribution. The resting variables had negligible influence on the yield of vanillin production at their individual levels and hence were considered not significant.

*Optimization using Box-Behnken design*

After screening of favorable factors, the Box-Behnken statistical design as one kind of the most effective response surface methods was applied to find the true optimum concentrations of four process independent variables, *i.e*. initial isoeugenol, initial NaCl, initial biomass and initial tween 80 for vanillin production. Table 4 and 5 show the details of the design matrix of the variables in the actual and coded units employed in the RSM along with the predicted and observed responses for vanillin yield. 

**Table 4:** Levels of the four independent variables used in Box-Behnken design experiments in terms of actual and coded factors

**Variables (g/L) **	**Actual**	**Coded**	**Actual**	**Coded**	**Actual**	**Coded**
Isoeugenol	4	-1	8	0	12	+1
Tween 80	0.4	-1	0.8	0	1.2	+1
NaCl	40	-1	80	0	120	+1
Dry Biomass	4	-1	8	0	12	+1

**Tabel 5:** Box-Behnken design matrix studies using four independent variables with three center points, showing observed and predicted vanillin concentrations

**Standard** **Order **	**Isoeugenol**	**Tween 80**	**NaCl**	**Dry Biomass**	**Vanillin yield (g/L)**	
					**Observed**	**Predicted**
1	-1	-1	0	0	1.53	1.48
2	+1	-1	0	0	1.79	1.78
3	-1	+1	0	0	1.07	1.11
4	+1	+1	0	0	1.87	1.96
5	0	0	-1	-1	1.79	1.73
6	0	0	+1	-1	1.34	1.37
7	0	0	-1	+1	1.29	1.25
8	0	0	+1	+1	2.11	2.16
9	-1	0	0	-1	1.56	1.63
10	+1	0	0	-1	1.89	1.89
11	-1	0	0	+1	1.46	1.47
12	+1	0	0	+1	2.43	2.37
13	0	-1	-1	0	1.32	1.40
14	0	+1	-1	0	1.12	1.07
15	0	-1	+1	0	1.43	1.44
16	0	+1	+1	0	1.69	1.58
17	-1	0	-1	0	1.73	1.75
18	+1	0	-1	0	1.78	1.83
19	-1	0	+1	0	1.53	1.53
20	+1	0	+1	0	2.58	2.61
21	0	-1	0	-1	1.40	1.37
22	0	+1	0	-1	1.04	1.04
23	0	-1	0	+1	1.28	1.29
24	0	+1	0	+1	1.39	1.43
25	0	0	0	0	2.15	2.14
26	0	0	0	0	2.19	2.14
27	0	0	0	0	2.18	2.14

For predicting our experimental results, the relationship of the independent variables and the response (Y) was estimated by a second order polynomial equation in the following form:

Y= *β*0+ *β*1X1+ *β*2X2+ *β*3X3+ *β*4X4+ *β*11X1^2^+ *β*22X2^2^+ *β*33X3^2^+ *β*44X4^2^+ *β*12X1X2+ *β*13X1X3+ *β*14X1X4+ *β*23X2X3+ *β*24X2X4+ *β*34X3X4           (Equation 2)

In this equation, Y is the predicted vanillin production yield; X1, X2, X3 and X4 represented the coded value concentrations of initial isoeugenol, initial tween 80, initial biomass, respectively (Tabel 4). *β*0 is the intercept coefficient; *β*1, *β*2, *β*3 and *β*4 are the linear coefficients; *β*11, *β*22, *β*33 and *β*44 are the quadratic coefficients; *β*12, *β*13, *β*14, *β*23, *β*24 and *β*34 are the interaction coefficients. The adequacy and significance of the above quadratic model was checked by several statistical criteria as presented in Table 6 and 7. Analysis of variance (ANOVA) of the response surface reduced quadratic polynomial model for vanillin production was performed to test the significance of the fit of the second-order polynomial Equation 2 as shown in Table 6. The very small p-value (< 0.0001) obtained from the analysis of ANOVA indicated that reduced quadratic polynomial model was highly significant and sufficient to represent the actual relationship between response (vanillin yield) and the significant variables (29). The Model F-value was 69.44 and the F-value for lack of fit was 12.88 (Table 6). 

**Table 6:** Analysis of variance of the response surface Reduced Quadratic Polynomial Model for vanillin production

**Source **	**Sum of Squares **	**Degrees of freedom (df) **	**Mean Square **	**F-value **	**p-value ** **Probability> F **
Model	4.32	13	0.33	69.44	<0.0001 significant
Isoeugenol	1.00	1	1.00	208.37	<0.0001
Tween 80	0.027	1	0.027	5.65	0.0334
NaCl	0.23	1	0.23	47.39	<0.0001
Biomass	0.074	1	0.074	15.38	0.0018
Isoeugenol × Tween 80	0.073	1	0.073	15.23	0.0018
Isoeugenol × NaCl	0.25	1	0.25	52.22	<0.0001
Isoeugenol × Biomass	0.10	1	0.1	21.39	0.0005
Tween 80 × NaCl	0.053	1	0.053	11.05	0.0055
Tween 80 × Biomass	0.055	1	0.055	11.53	0.0048
NaCl × Biomass	0.40	1	0.40	84.22	<0.0001
Tween 80 × Tween 80	1.88	1	1.88	392.41	<0.0001
NaCl × NaCl	0.27	1	0.27	56.37	<0.0001
Biomass × Biomass	0.55	1	0.55	115.31	<0.0001
Residual	0.062	134.788E-003			
Lack of Fit	0.061	115.580E-003		12.88	0.0742 not significant
Pure Error	8.667E-004	24.333E-004			
Cor Total	4.38	26			

The high F-value and non-significant lack of fit imply that the model is significant. Table 7 shows the backward elimination logistic regression analysis of the above model for the optimization of vanillin production. The coefficient of variation (CV) is very low (4.16), indicating a very high degree of precision and a good deal of reliability of the experimental values. The goodness fit of the model was checked by the determination coefficient (*R*^2^). 

**Table 7:** Backward elimination logistic regression analysis of the second-order polynomial model for the optimization of vanillin production

**Factor **	**Coefficient Estimate **	**Standard Error **	**95 % Cl ** **Low **	**95 % Cl ** **High **	**VIF **
Intercept	2.14	0.030	2.08	2.21	
Isoeugenol	0.29	0.020	0.25	0.33	1.00
Tween 80	-0.048	0.020	-0.091-4.347E-003		1.00
NaCl	0.14	0.020	0.094	0.18	1.00
Biomass	0.078	0.020	0.035	0.12	1.00
Isoeugenol × Tween 80	0.14	0.035	0.06	0.21	1.00
Isoeugenol × NaCl	0.25	0.035	0.18	0.32	1.00
Isoeugenol × Biomass	0.16	0.035	0.085	0.23	1.00
Tween 80 × NaCl	0.12	0.035	0.040	0.19	1.00
Tween 80 × Biomass	0.12	0.035	0.043	0.19	1.00
NaCl × Biomass	0.32	0.035	0.24	0.39	1.00
Tween 80 × Tween 80	-0.56	0.028	-0.62	-0.5	1.11
NaCl × NaCl	-0.21	0.028	-0.27	-0.15	1.11
Biomass × Biomass	-0.3	0.028	-0.36	-0.24	1.11

R^2^: 0.9858 Adjusted-R2: 0.9716 Predicted-R^2^: 0.9290 Adeq Precision: 31.510

Coefficient of variation (CV %): 4.16 PRESS: 0.31

**Figure 2:**
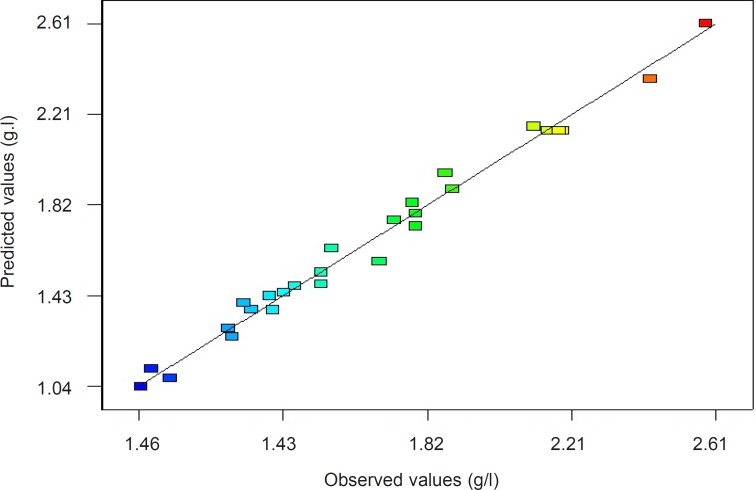
Parity plot showing the distribution of the observed response values versus the predicted response values of vanillin yield

In this case, the value of the determination coefficient (*R*^2^=0.9858) indicates that only 98.58% of the total variation in the vanillin yield is attributed to the independent variables. The adjusted R^2^ value of 0.9716 and Adeq Precision (the signal to noise ratio) of 31.51 was also satisfactory to confirm the significance of the model (30). The Parity plot indicated a satisfactory distribution between the observed response values versus the predicted response values (Figure 2), wherein, the points cluster around the diagonal line which shows the high dependence and correlation between the measured and the predicted values of response (31). By employing multiple regression analysis on the experimental data, the regression equation coefficients were estimated and the data was fitted to a second-order polynomial equation. The regression results are given in Table 7. Using the results of the experiments, the following second order polynomial equation (Equation 3) was established to explain the response, vanillin yield (Y, g/L) with *Psychrobacter *sp. CSW4 as a function of isoeugenol concentration (A, g/L), tween 80 concentration (B, g/L), NaCl concentration (C, g/L) and Biomass (D, g/L): 

R=+2.14+0.29A-0.048B+0.14C++0.078D+0.14AB+0.25AC+0.16AD+0.12BC+ 0.12BD+0.32CD-0.56B^2^-0.21C^2^-0.30D^2^                     (Equation 3) 

**Figure 3:**
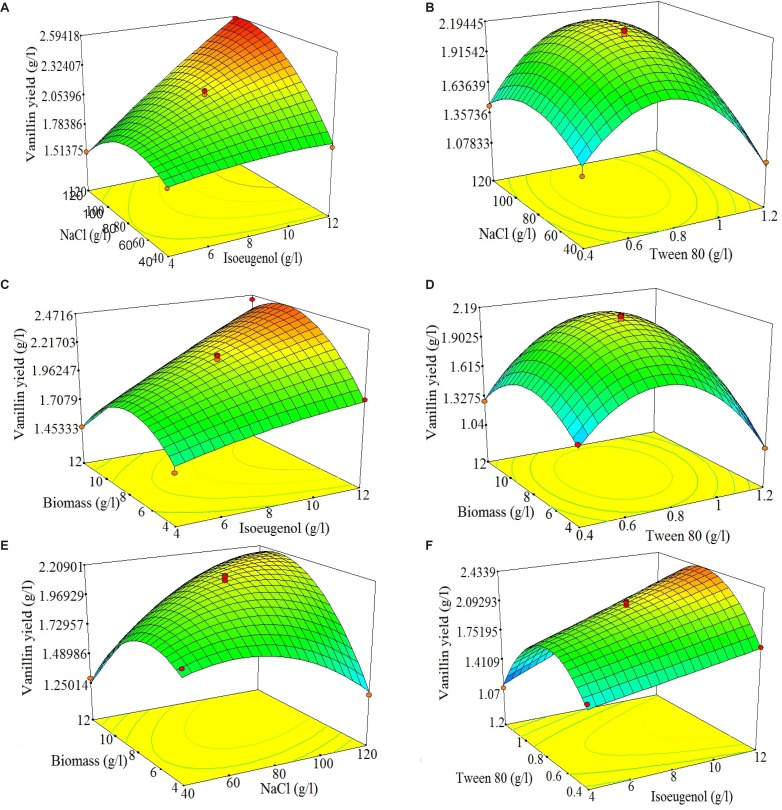
3D response plots and corresponding contour plots of vanillin production by *Psychrobacter *sp. CSW4 showing mutual interactions between different components (A: Isoeugenol vs. Tween 80; B: Isoeugenol vs. NaCl; C: Tween 80 vs. NaCl; D: Isoeugenol vs. Biomass; E: Tween 80 vs. Biomass; F: NaCl vs. Biomass). Other variables were at their respective zero levels

Using Design Expert software, the three-dimensional response surfaces are plotted in Figure 3 (A-F). The 3D response surface plots described by the regression model were drawn to illustrate the combined effects of isoeugenol-tween 80 (Figure 3A), isoeugenol-NaCl (Figure 3B), tween 80-NaCl (Figure 3C), isoeugenol-biomass (Figure 3D), tween 80-biomass (Figure 3E) and NaCl-biomass (Figure 3F). The response surface plots can be used to identify the major interactions between the test variables from the circular or elliptical nature of the contours (32). A circular contour plot shows that the interactions between related variables are negligible. An elliptical contour plot shows that the interactions between related variables are significant (29). By analyzing plots **(**Figure 3A-F), the predicted yield of vanillin was observed in the following ranges of the examined variables: isoeugenol 6.25-7.5 g/L, NaCl 110-120 g/L, tween 80 0.8-1 g/L and biomass 6.25-7.5. By analyzing the three-dimensional response surface plots and the corresponding contour plots, the optimized values of the independent variables could be predicted, but it was difficult to analyze all these simultaneously. Hence, point prediction of design expert software was used to determine the optimum values of the factors for maximum vanillin yield (33). By solving the Equation (3) using point prediction of above-mentioned software, the optimum values of isoeugenol 6.5 g/L, tween 80 0.89 g/L, NaCl 113.2 g/L and biomass 6.27 g/L were determined. These values predict 2.25 g/L of vanillin production. These optimized values of the factors were validated in a triplicate shakes flask study and an average 2.19 g/L of vanillin yield (molar yield of 36.3%) was obtained. This shows 97.3% validity of the predicted model. The good correlation between predicted and experimental values after optimization justified the validity of the response model and the existence of an optimum point. 

## Conclusion

Traditional methods of optimization are usually time consuming, labour-intensive processes and they cannot also explain the actual interactions of the factors of the experimental data and thus lead to misinterpretation of results that are used to select the significant parameters that influenced the process. A statistical approach in experimental design of biotechnological processes is confirmed to overcome the limitations of conventional optimization process and allows quick identification of the important factors and interactions between them (23). In the current work, the statistical methodology, combination of the Taguchi design and the Box-Behnken design under RSM is employed in selecting the statistically significant variables and finding the optimal concentration of those variables for maximizing vanillin production by the resting cells of *Psychrobacter *sp. CSW4 in a bioconversion process. 

Our results indicate the optimized process parameters for the bioconversion of isoeugenol by *Psychrobacter *sp. CSW4 might result in a significant reduction in the cost of medium constituents. The present work is the first to report on the application of Taguchi design and Response surface methodology for the optimization of culture conditions to improve bioconversion of isoeugenol into vanillin under resting cell conditions.
